# GGAs: Regulation of Multiple Sorting Pathways and Potential Association With Human Diseases

**DOI:** 10.1111/jcmm.71215

**Published:** 2026-06-03

**Authors:** Qinqin Wang, Juan Mei, Shuyue Li, Guoan Zhang, Jian Qiu, Xuezhi Li

**Affiliations:** ^1^ Shandong Collaborative Innovation Center for Diagnosis, Treatment and Behavioral Interventions of Mental Disorders, Institute of Mental Health Jining Medical University Jining China; ^2^ Jining Key Laboratory of Cognitive Impairment, Institute of Mental Health Jining Medical University Jining China; ^3^ Jining Key Laboratory of Forensic Multimodal Precision Identification, School of Forensic Medicine Jining Medical University Jining China; ^4^ Precision Medicine Laboratory for Chronic Non‐Communicable Diseases of Shandong Province, School of Forensic Medicine Jining Medical University Jining China; ^5^ Department of Neurology Affiliated Hospital of Jining Medical University Jining China

**Keywords:** Alzheimer's disease, cancer, Golgi‐localized gamma‐ear‐containing Arf‐binding protein, sorting adaptor, type 2 diabetes mellitus

## Abstract

Golgi‐localized gamma‐ear‐containing Arf‐binding proteins (GGAs) are a family of monomeric clathrin adaptors that function in intracellular vesicle trafficking. The three GGA family members—GGA1, GGA2 and GGA3—were first identified as sorting adaptors almost simultaneously by independent research groups in 2000. It is now well established that GGAs exert broad functions in intracellular sorting pathways. Moreover, GGAs have been shown to participate in diverse cellular processes including cell survival, migration and invasion, and are proposed to be potentially associated with multiple human disorders, such as Alzheimer's disease, cancers and type 2 diabetes mellitus. In this review, we briefly summarize the current knowledge regarding the roles of GGAs in intracellular vesicle trafficking, discuss the regulatory mechanisms underlying their functions, and speculate on their potential implications in human diseases. These findings may help us understand how GGAs participate in the pathogenesis of human diseases and, in the future, how to intervention the diseases via GGA‐targeted strategies.

## 1 Introduction

Golgi‐localized gamma‐ear‐containing Arf binding proteins (GGAs) comprise a family of proteins that function as Arf‐dependent clathrin adaptors in the assembly of the coat‐mediated trafficking machinery during intracellular vesicle formation and cargo selection [1, 2]. GGAs act as monomeric clathrin adaptors, which are recruited from the cytosol to membranes under the regulation of the ADP‐ribosylation factor (Arf) family of small GTP‐binding proteins to determine vesicle sorting fate [3, 4].

GGA proteins were initially identified as sorting adaptors involved in regulating the transport of Golgi‐derived vesicles almost simultaneously by independent groups in 2000 [2, 3, 4, 5, 6]. In 2001, these newly discovered GGA proteins were described as ‘new players in the sorting game’ in a review [1]. Further findings on GGAs were summarized in 2004 [7, 8]. Over the past two decades, our understanding of the structural basis of GGAs has advanced significantly, and novel functions of these adaptor proteins have been identified—particularly their role in endocytic trafficking. As the increasingly widespread sorting roles of GGAs are uncovered, the physiological functions of GGAs have attracted growing attention. GGAs have been found to be involved in processes such as cell survival, migration, and invasion, and their potential involvement of GGAs in human disorders is increasingly recognized.

It is now clear that GGAs are versatile players in the ‘sorting game’ [9]. Here, we briefly summarize historical findings as well as recent progress in understanding the sorting roles of GGAs, analyse the modulation of their function, and discuss their possible involvement in human diseases.

## 2 The Structural Basis of GGAs

**FIGURE 1:**
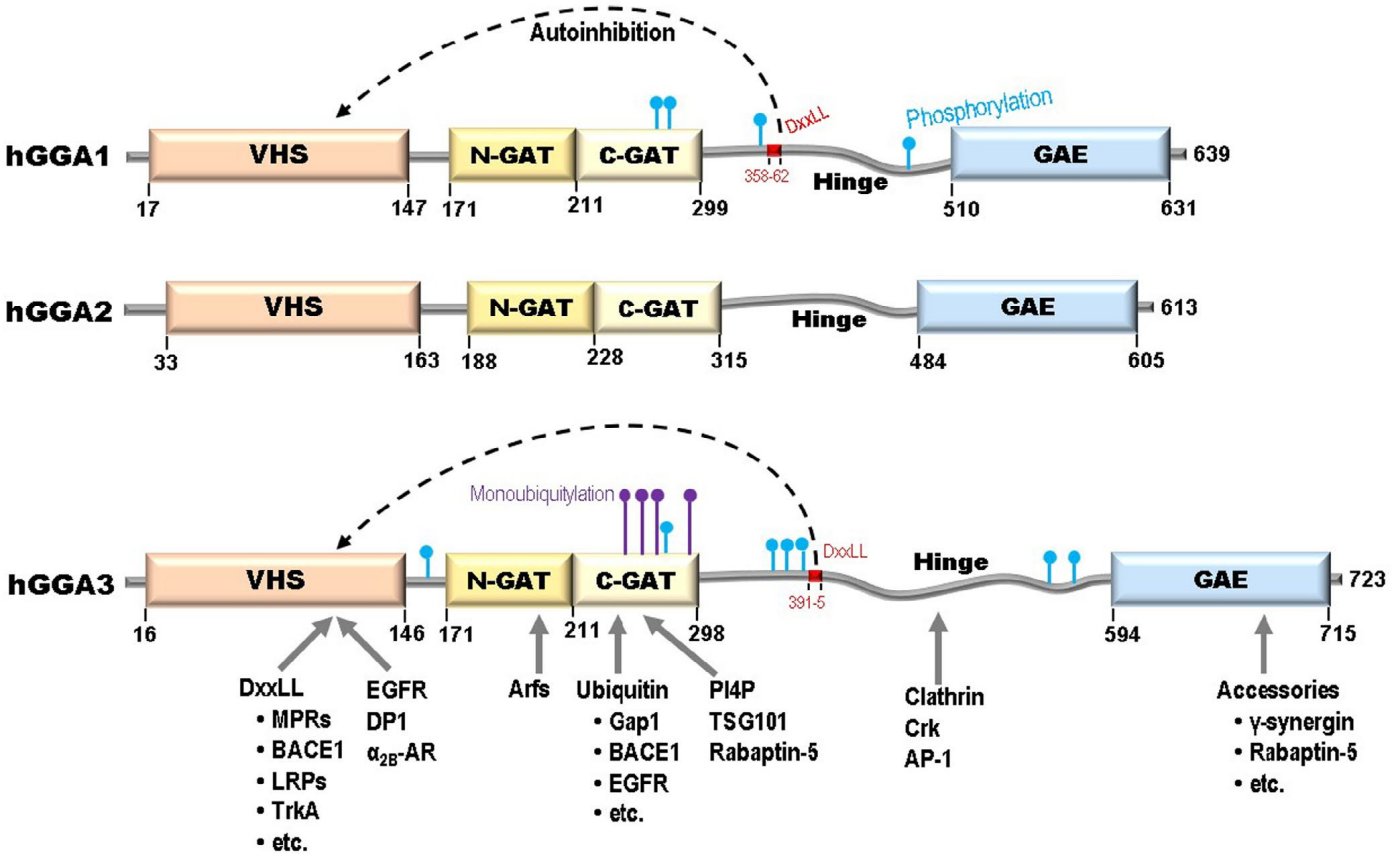
Structural features of GGAs (based on the sequence of human GGAs). The VHS domain, so‐called because of its occurrence in Vps27, Hrs, and STAM, consists of ~130 residues and is followed by the GAT (conserved in GGAs and TOM1) domain, comprising ~130 residues. The ‘hinge’ region is an unstructured region that contains clathrin‐binding motifs. The C‐terminal region of GGA proteins, containing ~120 residues, is known as the GAE (gamma adaptin ear) domain because of its homology to the ear domain of γ‐adaptin.

The three known GGA family members—GGA1, GGA2, and GGA3—share four conserved structural domains: the N‐terminal VHS domain, the GAT domain, the ‘hinge’ region, and the C‐terminal GAE domain (Figure 1) [2, 10, 11, 12, 13]. Briefly, the VHS domain mediates cargo interaction; the GAT domain is responsible for cargo interaction and Arf binding; the hinge region is primarily responsible for clathrin and adaptor protein‐1 (AP‐1) binding; the GAE domain is responsible for engages with accessory proteins. The interacting partners and accessory molecules of GGAs are summarized in Table 1.

**TABLE 1:** Partner and accessory molecules of GGAs.

Name	Interaction	Consequence
Arfs	GGA N‐GAT with active Arfs	Regulation of GGA localization [2, 3, 14, 15]
Clathrin	GGA hinge region and GAE domain with the terminal domain of clathrin	Clathrin recruitment to the TGN membrane [14]
AP‐1	GGA‐WNSF with AP‐1 γ‐ear	Cooperate in the packaging of MPRs into clathrin‐coated vesicles at the TGN [16, 17]
Rabaptin‐5	GAE or hinge‐GAE with FGxLV motif, GAT with C‐terminal coiled‐coils	Decreases the binding of clathrin to GGA, shifts the localization of endogenous GGA1 and associated cargo to enlarged early endosomes [18, 19]
TSG101	VHS‐GAT domains	Endosome sorting [19, 20]
γ‐Synergin	GAE domain	Localize in the TGN region [6]
p56	GAE with p56 DFGGF motif [21]	P56 cooperates with GGAs in the sorting of cathepsin D to lysosomes, promotes p56 localization to the TGN [21, 22, 23]
GAK	GGA1 GAE with GAK WAAWTE motif [21]	Unclear
PI4KIIIβ	GGA2 VHS [24]	Promote accumulation of PIP4P and recruitment of AP‐1 to the TGN [24, 25]
CLINT1	GAE domain	Recruitment to the TGN and endosomal clathrin coats [26, 27, 28]
Aftiphilin	GAE domain	Recruitment to theTGN and endosomal clathrin coats [27, 29, 30]
Crk	GGA3 binds Crk through two putative proline‐rich Crk SH3‐binding sites in the hinge region	MET endosomal recycling [31]
PACS1	GGA3 VHS‐GAT with PACS1 at 246‐AEIKIYSLSS	The localization of TGN membrane proteins that contain acidic cluster sorting motifs [32, 33, 34]
NHE6	NHE6 cytoplasmic tail with GGA1	The localization of intra‐cellular NHEs to the endosome compartment [35]

Abbreviations: AP‐1, adaptor protein 1; CLINT1, clathrin interactor 1, also known as EpsinR; GAK, cyclin‐G‐associated kinase; MET, Mesenchymal Epithelial Transition; NHE6, Sodium‐hydrogen exchanger 6; PACS1, phosphofurin acidic cluster sorting protein‐1; PI4KIIIβ, phosphatidylinositol‐4‐kinases III beta; PI4P, phosphatidylinositol‐4‐phosphate, also known as PtdIns4P; TSG101, tumour susceptibility gene 101 protein.

In terms of the common architecture of GGAs, the VHS domain is a right‐handed superhelix composed of eight helices with conserved hydrophobic residues [36, 37]. Early studies have shown that the VHS domain interacts with acidic dileucine motifs ( **D** xxLL) in cargo proteins during trans‐Golgi network (TGN) sorting and the endocytic trafficking of cell‐surface proteins [38, 39, 40]. Specifically, residues at positions −2 to 2 of the acidic cluster ( **D** xxLL, where residues are numbered relative to Asp 0) in cargo proteins interact most extensively with the N‐terminus of helix 6 in GGA3 [36]. Phosphorylation of Ser residues adjacent to the acidic cluster—such as Ser (−1) in the cation‐independent mannose 6‐phosphate receptor (CI‐MPR)—regulates the interaction between this cluster and the VHS domain of GGA proteins [41, 42, 43]. The cargo proteins sorted by GGAs, along with their corresponding sorting signals and sorting pathways, are summarized in Table 2.

**TABLE 2:** Sorting cargos of GGAs.

Name	Interaction	Sorting pathway
CI‐MPR	2479‐SFHDDS **D** ED **LL** HI ubiquitin	Bidirectional transport between the *trans*‐Golgi network (TGN) and endosomes [38]; from the TGN to the endosome and finally the lysosome [20]
CD‐MPR	265‐EESEER **D** DH **LL** PM	Bidirectional transport between the TGN and endosomes [39]
LRP3	758‐MLEASD **D** EA **LL** VC	TGN‐cell surface trafficking [44, 45]
LRP10	683‐LALEDE **DD** V **LLV** PLAEPG‐ 702‐WVAEAE **D** EP **LL** T	
LRP12	847‐KNETSD **D** EA **LL** LC	
Adiponectin	Adiponectin with VHS‐GAT of GGA1	Secretion [46]
Sortilin	41‐GYHDDS **D** ED **LL** E	Golgi‐endosome transport [47, 48]
SorLA/LR11	41‐ITGFSD **D** VP **MV** IA	Recycling [49]
Stabilin‐1	2510‐FSAFQAEDDADDDFSPWQE 2546‐TFCEPF **D** DS **LL**	TGN to endosome and sorting [50] intracellular sorting [51]
ST7	330‐KNETSD **D** EA **LL** LC	Unclear
BACE1	490‐HDDFAD **D** IS **LL** K Ubiquitin	Endosomes to the TGN, degradation, recycling, axonal transport [52, 53, 54, 55, 56]
TRiCβ	445‐HRVSQD **D** LD**LL**TS	Unclear
CLCN7	59‐MSSVELDDELLDPDMDP—	Lysosome location [57]
Consortin	DxxLL	Plasma membrane targeting and recycling of connexins [58]
RNF11	8‐PTSD **D** IS **LL** HESQ‐20 141‐EPV **D** AA **LL** SSYET‐153	RNF11 sorting at the TGN and its internalization from the plasma membrane [59]
IR	Unclear	Regulation of IR endocytic trafficking [60]
TrkA	531‐ NLLPEQDKMLVAVKALK—	Endocytic recycling [40]
RET	791‐YGACSQDGPLLLIVEYA—	Endocytic recycling [61]
Gap1	Ubiquitin	Golgi → endosome → lysosome [62]
LAPTM5	LAPTM5‐UIM to ubiquitin‐GGA3	Transport of LAPTM5 to lysosomes [63]
EGFR	Juxtamembrane region with VHS‐GAT of GGA1, 2, and 3; ubiquitin with GGA3 GAT	Ubiquitin: lysosomal degradation [20]; juxtamembrane region: GGA1/GGA3 for lysosomal degradation, GGA2 for sustaining receptor expression [64]
DP1	Intracellular loop 2 (ICL2) and the C‐terminal tail (C‐tail) with GGA3 VHS	Endocytic recycling [65]
α2B‐AR	Intracellular loop 3 (ICL3) with GGA3 VHS	Surface expression [66, 67]
Adiponectin	Adiponectin with VHS‐GAT of GGA1	Secretion [46]
β1‐Integrin	Arf‐dependent	Diverts integrins from the degradative pathway [68]

Abbreviations: BACE1, β‐site amyloid precursor protein cleaving enzyme 1; CD‐MPR, cation‐dependent mannose‐6‐phosphate receptor; CI‐MPR, cation‐independent mannose‐6‐phosphate receptor, also known as IGF2 receptor and CD222; CLCN7, chloride voltage‐gated channel 7; DP1, prostaglandin D2 receptor; EGFR, epidermal growth factor receptor; IR, insulin receptor; LAPTM5, lysosomal‐associated protein transmembrane 5; LPR, low‐density lipoprotein receptor‐related protein; RET, proto‐oncogene tyrosine‐protein kinase receptor Ret; RNF11, Ring Finger Protein 11; ST7, suppressor of tumorigenicity 7 protein; TRiCβ, tailless complex polypeptide 1 ring complex β, also known as MARVEL domain containing 2, DFNB49, FLJ30532, and MRVLDC2; TrkA, tropomyosin‐related kinase A; α2B‐AR, alpha‐2B adrenergic receptor. The underline charactors indicates the key binding sites.

The GAT domain of GGAs can be divided into two subdomains: an N‐terminal helix–loop–helix motif (N‐GAT) containing a hydrophobic surface patch that directly interacts with the GTP‐bound forms of Arfs, and a C‐terminal three‐helix bundle (C‐GAT) primarily responsible for ubiquitin binding [69]. Although the GAT domain binds to Arf and ubiquitin via distinct subdomains, binding of Arf‐GTP to the N‐GAT subdomain has been shown to enhance the interaction between the C‐GAT subdomain and ubiquitin [69]. In addition to the acidic clusters and ubiquitin tags in cargo proteins, noncanonical interaction targets for GGAs also exist. The cytoplasmic juxtamembrane region of the epidermal growth factor receptor (EGFR) can interact with the VHS‐GAT domains of all three GGAs in a manner dependent on N108 in the VHS domain [64]. GGA3 binds to Crk (CT10 regulator of kinase) adaptor proteins through two putative proline‐rich Src Homology 3 (SH3) domain‐binding sites in the hinge region [31]. Interestingly, an intracellular loop (ICL) of α2B‐AR binds directly to the variable domains of the three GGAs [66, 67]. For specific targeting of GGAs and recognition of ubiquitinated cargo at the TGN, GGAs can interact with phosphatidylinositol 4‐phosphate (PI4P) via the C‐GAT subdomain [24].

The hinge region of GGA proteins also binds clathrin and interacts with the γ‐ear domain of AP‐1 [17]. Meanwhile, the GAE domain recruits accessory proteins by binding to a hydrophobic motif [13]. The GAT and GAE domains coordinately interact with the rabaptin‐5‐rabex‐5 complex: the GAT domains bind to the C‐terminal coiled‐coils of rabaptin‐5, while the GAE domains recognize an FGxLV sequence within the same protein [18, 70, 71]. Interestingly, the GGA1‐GAE domain has been proposed to bind to the WNSF sequence (residues 382–385) in the hinge region of GGA1, thereby mediating the autoregulation of GGA function by competing with accessory proteins and the AP‐1 complex [72].

## 3 The Sorting Pathways in Which GGAs Participate

GGA proteins were originally identified as clathrin adaptors complementary to adaptor protein complexes (APs) for clathrin‐coated vesicles (CCVs) [2, 3]. They were proposed to prime membranes for clathrin recruitment, with AP complexes subsequently intercalated into the forming coats [14]. The functional relationships between GGAs and AP‐1 in CCV formation may differ across distinct trafficking pathways: GGAs and AP‐1 can regulate CCV formation either independently or sequentially [9]. A very recent study using live‐cell imaging with simultaneous capture emphasized that GGAs and AP‐1 act independently, as GGA2 and AP‐1 puncta were mainly on separate trajectories, and no clear individual spots were observed transitioning from GGA2‐positive to AP‐1‐positive [73].

**FIGURE 2:**
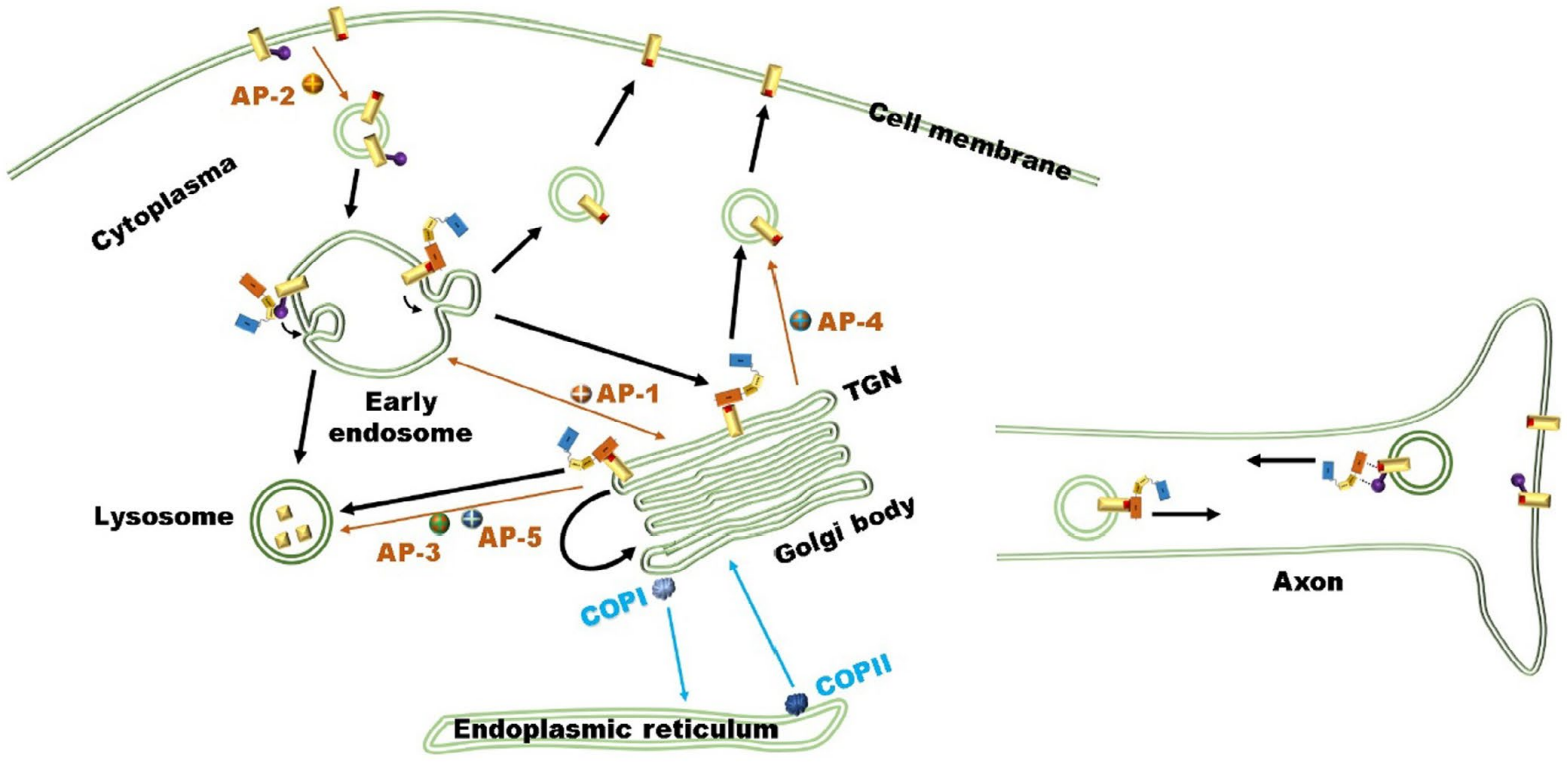
The known trafficking pathways served by GGAs (black arrow). The pathways APs (brown arrow) and COPs (blue arrow) participated are shown for comparation.

The cargo proteins sorted by GGAs and their corresponding sorting pathways are summarized in Table 2 and illustrated in Figure 2. The first confirmed cargo proteins of GGAs were mannose‐6‐phosphate receptors (MPRs), including the cation‐dependent mannose‐6‐phosphate receptor (CD‐MPR) and the cation‐independent mannose‐6‐phosphate receptor (CI‐MPR). MPRs traffic bidirectionally between the trans‐Golgi network (TGN) and lysosomes to transport lysosomal hydrolases [38, 74]. GGAs were subsequently found to be involved in trafficking among the TGN, endosomes, and lysosomes [48, 50, 74, 75]. A recent study suggested that GGAs are required for Vps10 trafficking from the prevacuolar endosome to the TGN [76].

GGAs have also been implicated in trafficking between the TGN and the cell surface. LDL receptor‐related protein 9 (LRP9 in mice, LRP10 in humans) is a member of the lipoprotein receptor family, predominantly localized to the TGN and endosomes. GGA proteins were reported to interact with LRP9, thereby facilitating its trafficking from the TGN to the cell surface and endosomes [44]. Moreover, GGA3 deficiency led to a reduction in the cell surface levels of α2B‐AR. Specifically, in HEK293 cells, knockdown of GGA3 only inhibited the transport of newly synthesized α2B‐AR from the TGN to the cell surface, whereas the total protein level, synthesis, and internalization of α2B‐AR remained unaffected [66]. Another study demonstrated that GGA1 and GGA2 regulate the cell surface levels of α2B‐AR in cortical neurons [67].

Two groups independently identified the binding of GGAs to ubiquitin almost simultaneously [20, 62]. Thereafter, additional instances of GGA‐regulated endocytic sorting were reported. Studies have shown that depletion of either GGA1 or GGA3 increases EGFR expression, which is proposed to result from disrupted endosomal sorting to the lysosomal degradation pathway. In contrast, GGA2 depletion significantly reduces the steady‐state expression of EGFR due to impaired recycling, indicating that the function of GGA2 in endosomal sorting differs from that of GGA1 and GGA3 [64]. To date, GGAs have been implicated in the endosomal sorting of BACE1 (degradation and recycling) [55, 77, 78, 79, 80], MET (recycling) [31, 81, 82], TrkA (recycling) [40], RET (recycling) [61], DP1R (recycling) [65], β1‐integrin (recycling) [83], and lipoprotein receptor LR11 (recycling) [49], among others.

GGAs also participate in axonal transport. Axonal transport is essential for maintaining neuronal homeostasis by delivering organelles, proteins, lipids, and RNAs from the neuronal soma to terminals, as well as clearing unwanted substances such as misfolded proteins and protein aggregates [84]. Impaired axonal transport has been linked to the development of various neurological diseases [84, 85]. A recent study by Lomoio et al. demonstrated that GGA3 localizes to both axons and dendrites and is co‐transported with BACE1 in axons [53]. Furthermore, GGA3 deficiency inhibits BACE1 axonal motility, leading to the accumulation of BACE1 in axonal swellings [53].

In summary, the known functions of GGA proteins have expanded from their originally identified role as TGN sorting adaptors to key regulators of endosomal degradation, recycling, and axonal transport. Clarifying the determinants of GGA function will advance our understanding of how to modulate the associated sorting processes and their related physiological outcomes.

## 4 Regulation GGAS' Sorting Functions

For the regulation of GGAs' sorting functions, less is known about how the gene expression is controlled and the upstream pathway modulating GGAs' activity. As an Arf protein dependent sorting adaptor, the recruit of GGAs to vesicle membrane and the formation of soring complex is regulated by upstream signal controlled activation of Arf proteins (transform between Arf‐GDP and Arf‐GTP) [40, 86, 87]. Such as a very recent study suggests a novel regulating flow for cell surface receptor to control the formation of GGA3 soring machinery, in which the ErbB3 (HER3) receptor promotes assembly of the Arf6–GGA3‐Rabaptin5 endosomal sorting complex to facilitate early recycling of Integrin β1 and suppress Integrin β1‐containing extracellular vesicle (EV) release [86]. However, studies suggest post‐translational modifications, such as ubiquitination and phosphorylation, are critical for regulating the interaction of GGAs with cargos and sorting partners and the degradation of GGAs. Several ubiquitination and phosphorylation sites in GGAs have been reported (Figure 1).

Soon after the identification of GGAs, it was found that GGA1 and GGA3 exhibit autoinhibition when binding to the DxxLL sequence of cargos [88]. Compared with full‐length versions, truncated forms of GGA1 and GGA3 (GGA1^1–240^ and GGA3^1–241^) showed an enhanced capacity to bind DxxLL‐containing cargos. An internal DxxLL motif was then identified in GGA1 and GGA3 (^358^DDELM^362^ in human GGA1 and ^391^DEELL^395^ in human GGA3) as the binding target of the VHS domain, preventing the binding of the VHS domain with DxxLL sequence of cargos. The phosphorylation state of a serine residue (Ser^355^ in GGA1) upstream of the acidic residues controls the autoinhibitory events. Specifically, when Ser^355^ is phosphorylated by protein kinase casein kinase 2 (CK2), GGA1 is localized to the cytosol, whereas dephosphorylation is associated with membrane recruitment. In another study, a PACS1/CK2‐stimulated phosphorylation cascade induced the phosphorylation of GGA3, thereby releasing it from CI‐MPR and early endosomes [34]. Although autoinhibition via internal DxxLL binding has been questioned [89], increasing evidence supported that the function of GGAs is subject to modulation by autoinhibition and the state of phosphorylation of the proteins [34, 42, 90, 91, 92, 93]. The complete protein sequence of GGA2 is 45% and 35% identical to those of GGA1 and GGA3, respectively [4]. However, GGA2 has no internal DxxLL motif and is thus not subject to autoinhibition [88].

In addition to the regulation of GGA activity through autoinhibition via the phosphorylation of serine and threonine residues near the internal DxxLL sequences, the phosphorylation of specific residues also affects the capacity of GGA proteins to bind target cargos by altering their conformation [42]. Epidermal growth factor (EGF)‐induced phosphorylation of S368 and S372 in GGA3 results in a hinge‐specific conformational change, which decreases its ubiquitin‐binding capacity and impairs its membrane association [91]. Hepatocyte growth factor (HGF) can induce GGA3 phosphorylation via PKCε, thereby perturbing its normal function in the early endosome and facilitating c‐Met degradation [92]. Thus, phosphorylation of GGAs may works as a feedback regulation for cargo sorting. Multiple serine and threonine residues in GGAs that can be phosphorylated have been identified, including S268, T270, S355, and S480 in human GGA1 and S159, S267, S368, S372, S388, S538 and S559 in the long isoform of human GGA3 [88, 91, 93]. GGA2 can also be phosphorylated, and several serine and threonine residues in GGA2 are identical to the reported phosphorylatable sites in GGA1 and GGA3. The exact residues that undergo phosphorylation require further investigation.

Ubiquitination also plays a regulatory role in GGA functions. GGAs are monoubiquitylated by human homologue of vacuolar protein sorting 18 (hVPS18), a RING‐H2 type ubiquitin ligase [69, 94]. A lysine residue in the C‐terminal of the GAT domain (K258 in GGA3) was identified as a major ubiquitylation site, while other lysine residues (K249, K264, K294) were also found to be ubiquitylated to some extent by hVPS18 [94]. The E3 ubiquitin‐ligase NEDD4 can ubiquitinate GGA3 to promote the formation of NEDD4/LAPTM5/GGA3 complex through the interaction between LAPTM5‐UIM and ubiquitin‐GGA3, and finally drive the sorting of LAPTM5 from the Golgi to the lysosome [63]. Ring finger protein 11 (RNF11) can recruit itch to drive the ubiquitination of GGA3 and promote GGA3 degradation [59]. Indeed, RNF11 has two DxxLL motifs recognized by GGAs and is a cargo sorted by GGAs at the TGN and its internalization from the plasma membrane [59]. Thus, ubiquitination of GGAs may also works as a feedback regulation for cargo sorting. Interestingly, GGA monoubiquitylation requires prior binding to ubiquitin tags of cargo, while GGAs can no longer bind these ubiquitin tags when monoubiquitylated [94].

## 5 The Expression and Biological Function of GGAs

The expression of human GGAs as estimated by the GeneCards online database exhibits a tissue‐ and developmental stage‐specific pattern (https://www.genecards.org/). However, relatively few studies have reported on the exact expression patterns of these proteins. In the progress of mouse development, *Gga1* and *Gga3* show increasing expression trends from embryonic to adult stages [95]. In contrast, *Gga2* shows decreasing expression trends from embryonic to adult stages. The high expression of *Gga2* in early embryonic stages indicates that *Gga2* is more important for development. Indeed, *Gga2* null mice exhibit low birthweights and hypoglycemia and lethal within 3 weeks of birth, while the loss of either *Gga1* or *Gga3* leads to no obvious developmental deficit [95, 96]. Among the tissues assessed in adult mice, the protein levels of GGA1 were high in the brain, fat and lung, those of GGA2 were uniquely high in the fat, and those of GGA3 were uniquely high in the brain [95]. Interestingly, a study on *Drosophila* showed GGA was mainly expressed in the head flies, while there is also a gender‐specific increased expression in the testes of male flies [97].

Along with the reveal of the widespread sorting pathways participated by GGAs, the cellular and physiological functions of GGAs are attracting more attentions. Studies showed that GGAs are related to spermatogenesis both in Drosophila and mice (GGA1 in mince) [97, 98]. A very recent multiomics combined machine learning computational framework identified GGA1 as a programmed cell death related gene which is involved in permatogenesis [99]. GGA1 mRNA expression was upregulated during myogenesis in C2C12 cells, a murine myoblast cell line. GGA1 was found to regulate the formation and maturation of myotubes likely by targeting the endocytic insulin receptor (IR) to the cell surface [60]. GGA1 was also suggested to facilitate adiponectin secretion in both 3T3L1 adipocytes and HEK293 cells [46]. *Gga1* was recently suggested to facilitate the osteogenic differentiation of BMSCs in anterior cruciate ligament reconstruction (ACLR) mouse models, whereas total flavonoids of Rhizoma drynariae (TFRD) targeted oestrogen related receptor α (ESRRA) and oestrogen related receptor β (ESRRB) to transcriptionally activate Gga1 expression, which enhanced the viability and osteogenic differentiation of TFRD‐induced bone mesenchymal stem cells (BMSCs) [100]. Using data from a genome‐wide association study (GWAS) of spontaneous and isoprenaline‐stimulated lipolysis in the unique GENetics of Adipocyte Lipolysis (GENiAL) cohort, GGA3 (SNP: rs9909443) was identified as a candidate gene regulating lipolysis, thereby potentially participating in the genetic control of body fat [101]. This was subsequently functionally validated via small interfering RNA (siRNA)‐mediated knockdown in adipose‐derived stem cells [101].

Coincidently with the mentioned expression patterns and related cellular functions above, accumulating laboratory and clinical data indicates the potential roles of GGA proteins in human diseases (summarized in Table 3), typically Alzheimer's disease (AD), Cancers and Diabetes.

**TABLE 3:** Potentially associated disorders of GGAs.

Disorder name	Alteration of GGAs	Potential or predicted roles
AD	Deceased expression of GGA3 in AD brain [78, 102, 103] rs117805695, rs146877619, rs52809447, rs141316552, rs139958143, rs34008167, rs3178020, rs150787028 in GGA3 [53]	GGA1 and GGA2 promote BACE1 recycling [52, 104] GGA1 regulates BACE1 retrograde transport of from early endosome to TGN^112^, promotes APP endocytic recycling [49] GGA3 drives BACE1 degradation [55], regulates BACE1 axonal transport [53].
PD	Decreased GGA3 protein level [105]	Drive α‐synuclein oligomerization and facilitate α‐syn oligomer secretion [105]
Neuropathic pain	Decreased GGA1 expression in the hippocampus of rats model with electroacupuncture‐induced neuropathic pain [106]	Unclear
IOP	rs52809447 in *GGA3* [107, 108]	Unclear
T2DM	Decreased GGA1 expression in mast cells derived from skin tissue of T2DM patients [109] rs78338345 in *GGA3* [110, 111]	Targeting of synthesized GLUT4 to the insulin‐responsive GSC [112, 113]
HCCs	Upregulated GGA2 expression [64]	Promotes EGFR expression [64]
Colorectal cancers	Upregulated GGA2 expression [64]	Promotes EGFR expression [64]
Lung cancer	Upregulated GGA2 expression [114]	Promotes EGFR expression [114]
NSCLC	Decreased GGA2 expression [115]	Unclear
MIBC	Unclear	*GGA1* gene associates with survival outcomes of MIBC [116]
NOA	Decreased expression of GGA1 [99]	GGA1 linkes to spermatogenesis [99]

Abbreviations: AD, Alzheimer's disease; BACE1, β‐site amyloid precursor protein cleaving enzyme 1; EGFR, epidermal growth factor receptor; GLUT4, Glucose transporter 4; GSC, storage compartment; HSGS, Hepatocellular carcinomas; IOP, Intraocular pressure; MIBC, Muscle‐invasive bladder cancer; N/A, not applicable; NOA, Non‐obstructive azoospermia; NSCLC, Non‐small cell lung cancer; PD, Parkinson's disease; T2DM, Type 2 diabetes mellitus.

## 6 The Potential Association of GGAs With AD

The most well‐characterized disease potentially associated with GGAs is AD. AD is a neurodegenerative disease with a high incidence among older adults. Several studies have reported decreased expression of GGA3 in the brains of AD patients [78, 102, 103]. Furthermore, genetic association analysis based on 3 whole‐genome sequencing (WGS) and 1 whole‐exome sequencing (WES) online AD datasets identified 7 suspected AD‐associated single nucleotide polymorphisms (SNPs) (rs117805695, rs146877619, rs52809447, rs141316552, rs139958143, rs34008167, rs3178020) and 1 suspected small insertion/deletion (INDEL) (rs150787028) in *GGA3* [53]. Thus, decreased expression of GGA3 in the temporal cortex of AD patients may contribute to the overproduction of amyloid‐β (Aβ) and the impairment of nerve growth factor (NGF) neurotrophic signalling—both of which are considered key pathological mechanisms of AD. It is noteworthy to understand how GGAs exert multifaceted roles in regulating the trafficking of β‐site amyloid precursor protein‐cleaving enzyme 1 (BACE1) and NGF receptors, as this may provide insights into the pathogenesis and targeted treatment of AD.

The involvement of GGAs in AD and the underlying mechanisms require further exploration. The major neuropathological hallmarks of AD include the presence of amyloid plaques composed of Aβ peptides and the selective degeneration of basal forebrain cholinergic neurons (BFCNs). The pathogenesis of AD remains incompletely understood, and disease‐modifying treatments for AD are currently lacking. The amyloid cascade hypothesis of AD proposes that Aβ oligomers are the primary cause of the disease [117], while the neurotrophic factor hypothesis suggests that deficiencies in neurotrophic signalling contribute to AD pathogenesis [118, 119].

Aβ peptides are proteolytic fragments of amyloid precursor protein (APP) and are generated via the β‐secretase‐mediated amyloidogenic pathway [120, 121]. BACE1 is the major β‐secretase and serves as the rate‐limiting enzyme in this pathway. He et al. first identified the interaction between GGAs and BACE1, with GGA1 and GGA2 binding to BACE1 to promote its recycling from early endosomes to the cell surface [52, 104]. GGA1 was subsequently found to regulate the retrograde transport of BACE1 from early endosomes to the TGN [54]. Additionally, GGA1 mediates the endocytic trafficking of LR11 to facilitate APP transport to the endocytic recycling compartment, thereby preventing Aβ production [49].

Furthermore, GGA3 directs BACE1 from early endosomes to lysosomal degradation [55]. Notably, GGAs are involved in multiple trafficking routes of BACE1 from early endosomes, including recycling, retrograde transport, and degradation—processes believed to efficiently remove BACE1 from endosomes and prevent Aβ overproduction [114, 115, 122, 123, 124]. Moreover, GGAs interact with BACE1 through distinct mechanisms in these trafficking routes: binding to the DxxLL motif of BACE1 for recycling, interacting with phosphorylated BACE1 for retrograde transport, and binding to the ubiquitin tag of BACE1 for degradation [114, 115, 122, 123, 124]. Recently, GGA3 was found to participate in the axonal transport of BACE1 [53]. Depletion of the *Gga3* gene and a rare small INDEL variant (rs150787028) of GGA3 have been shown to impair BACE1 axonal transport, consequently leading to Aβ overproduction [53]. GGA3 also regulates the NGF pathway by enhancing TrkA post‐endocytic recycling [40] or rapidly recruiting the p75 neurotrophic receptor (p75NTR) to the plasma membrane following TrkA activation by NGF^113^.

## 7 The Potential Association of GGAs With Cancers

Evidence supporting the involvement of GGAs in cancer pathogenesis has primarily accumulated in recent years. GGA2 has been implicated in hepatocellular carcinoma (HCC), colorectal cancer, lung tumorigenesis, and breast cancer. GGA2 protein expression is significantly upregulated in tumour regions of HCC tissues [64]. Tissue microarray analysis showed that 30.8% of HCCs and 23.3% of colorectal cancers exhibit increased GGA2 expression. Although all three GGA isoforms interact with the epidermal growth factor receptor (EGFR), GGA1 and GGA3 downregulate EGFR protein levels, while GGA2 promotes EGFR expression. Thus, the elevated GGA2 levels observed in a subset of HCCs and colorectal cancers may be attributed to its role in supporting cell proliferation by interacting with EGFR to sustain receptor expression [64]. Through integrative genomic analysis, GGA2 was further identified as a cooperative driver of EGFR‐mediated lung tumorigenesis [114]. In EGFR‐mutant lung adenocarcinoma (LUAD), overexpression of GGA2 enhances EGFR‐mediated transformation, whereas GGA2 knockdown reduces colony formation and tumorigenic potential [114]. Additionally, GGA2 maintains EGFR cell surface levels in H1975 cancer cells by directing the recycling of endocytosed EGFR, thereby sustaining cancer cell proliferation [82].

In non‐small cell lung cancer (NSCLC), GGA2 expression is decreased in tumour tissues, and exogenous overexpression of GGA2 in NSCLC cell lines inhibits cellular proliferation [115]. GGA2 has also been recognized as a favourable prognostic factor for NSCLC [115]. Conversely, another study reported increased GGA3 expression in NSCLC tissues and cell lines [123]. In the human NSCLC cell line A549, GGA3 overexpression promotes cell proliferation, invasion, and migration while inhibiting apoptosis [123]. These effects of GGA3 upregulation are proposed to occur via activation of the TrkA‐AKT/ERK signalling pathways [123], consistent with our previous finding that GGA3 promotes TrkA recycling to maintain TrkA levels and downstream Akt signalling [40].

GGA3 has also been shown to promote the endocytic recycling of RET51, sustaining RET51 surface expression and RET‐mediated AKT activation, which ultimately contributes to RET51‐dependent cell motility, migration, and invasion in SH‐SY5Y neuroblastoma cells [61]. Depletion of GGA2 reduces the cellular levels of other tyrosine kinases (MET and ErbB4), thereby suppressing the growth of H1975 cancer cells in both in vitro cultures and xenograft models [82]. Since GGA2 regulates the recycling of active β1‐integrins, silencing GGA2 decreases active β1‐integrin levels in focal adhesions and impairs the migratory and invasive potential of breast cancer cells [83]. Additionally, GGA3 is involved in the migration of breast epithelial cell sheets by regulating the early recycling of Integrin β1 and suppressing the release of Integrin β1‐containing extracellular vesicles (EVs) [86].

GGA1 participates in Golgi retention and oncogenic KIT signalling in gastrointestinal stromal tumour (GIST) cells [124, 125]. KIT belongs to the class III receptor tyrosine kinases (RTKs), and most GISTs arise from gain‐of‐function mutations in the KIT gene. Mutant KIT is retained in the Golgi/trans‐Golgi network (TGN) during biosynthetic transport, where it activates downstream signalling molecules, including the phospholipase Cγ2–protein kinase D2–PI4 kinase IIIβ (PLCγ2–PKD2–PI4KIIIβ) pathway. This activated signalling cascade aberrantly recruits GGA1 and the γ‐adaptin subunit of AP1, resulting in the retention of mutant KIT in the Golgi/TGN^118^. A very recent study combining nanopore sequencing and machine learning approaches revealed that *GGA1* may be involved in the pathogenesis of urothelial carcinoma and hold potential as prognostic biomarkers for muscle‐invasive bladder cancer (MIBC) patients [116].

## 8 The Potential Association of GGAs With Type 2 Diabetes Mellitus

Yasukochi et al. identified a SNP (rs78338345) in *GGA3* as a novel susceptibility locus for type 2 diabetes mellitus (T2DM) using longitudinal exome‐wide association studies in a Japanese population [110, 111]. Govero et al. found that newborn mice with *Gga2* deletion exhibited hypoglycemia (low blood glucose levels) [95]. In humans, neonatal hypoglycemia (either asymptomatic low blood glucose or symptomatic hypoglycemia) typically persists for a few hours to days after birth, followed by the maintenance of normal blood glucose levels. This phenomenon is thought to result from relative fetal hyperinsulinism, which balances the high glucose levels induced by maternal diabetes [126]. Thus, the lower blood glucose levels observed in newborn *Gga3* knockout (*Gga3* ^−/−^) mice might also reflect maternal diabetes or elevated glucose levels in adult mice. Recently, we found that young *Gga3* knockout (*Gga3* ^−/−^) C57BL/6J mice have elevated fasting blood glucose levels, indicating that GGA3 plays a protective role in glucose homeostasis [127]. A transcriptional analysis of skin‐derived mast cells from patients with T2DM indicated that GGA1 is downregulated in these cells [109]. Collectively, these findings suggest that GGAs may exert a protective role in T2DM.

Early studies suggested that GGAs regulate the trafficking of facilitated glucose transporter member 4 (GLUT4) vesicles [112, 113]. GLUT4 is a glucose transporter responsible for postprandial glucose clearance in response to insulin stimulation. Under basal conditions, GLUT4 is sequestered in GLUT4 storage compartments (GSCs) and redistributes to the plasma membrane following insulin stimulation—an essential process for glucose uptake [112]. GGAs are responsible for targeting de novo‐synthesized GLUT4 to insulin‐responsive GSCs both in vitro and in 3 T3‐L1 adipocytes, likely through interacting with GLUT4 vesicles via sortilin [112, 113]. Depletion of *Gga1* in the murine C2C12 cell line was shown to impair the formation of mature myotubes [60]. The authors proposed that GGA1 influences insulin‐dependent glucose uptake in C2C12 myotubes by regulating the endocytic trafficking of the IR; GGA1 deletion leads to increased lysosomal degradation of IR [60].

## 9 Hints of the Potential Association of GGAs With Other Disorders

### 9.1 Parkinson's Disease (PD)

GGA3 has been demonstrated to induce α‐synuclein (α‐syn) oligomerization in endosomal compartments and facilitate α‐syn oligomer secretion, ultimately exacerbating extracellular α‐syn toxicity in N2A cells [105]. The aggregation of α‐syn and its resulting cytotoxicity are hallmarks of PD and Lewy body dementia. Interestingly, no changes in GGA3 mRNA levels were detected in the post‐mortem substantia nigra (SN) of PD patients, whereas GGA3 protein levels were decreased—likely due to the loss of dopaminergic neurons and the small sample size (*n* = 2).

### 9.2 Intraocular Pressure (IOP)

Intraocular pressure (IOP), a major pathological feature of glaucoma, is associated with optic nerve neurodegeneration. Genetic analysis using bioinformatics tools has also suggested *GGA3* as a susceptibility locus for IOP (SNP: rs52809447) [107, 108].

### 9.3 Neuropathic Pain

*Gga1* has also been suggested to be associated with neuropathic pain [106]. Most patients with peripheral neuropathic pain are accompanied by cognitive impairments, which are thought to be related to structural and functional abnormalities in the hippocampus [128, 129, 130]. GGA1 expression was found to be downregulated in the hippocampus of a rat model of electroacupuncture‐induced neuropathic pain [106].

### 9.4 Non‐Obstructive Azoospermia

In a very recent study, GGA1 was suggested to be a hub gene for Non‐obstructive azoospermia (NOA) [99]. As been mentioned above, *GGA1* was identified as a programmed cell death related gene which is involved in permatogenesis, and the change in *GGA1* expression may lead to the male reproductive system disorders [99]. Consistently, *Gga1* had a decreased expression in the NOA model mice [99]. A very recent GWAS study in Duroc Boar also identified *Gga1* as candidate gene being significantly associated with sperm freezing tolerance [131].

### 9.5 Indirectly Associated Disorders

Two recent studies have reported that mutations in binding partners of GGAs are pathogenic, as they disrupt the normal interaction with GGAs [33, 132]. A novel *PACS1* variant (NM_018026.4:c.755C>T) that impairs GGA3 binding predisposes individuals to syndromic intellectual disability, indicating a potential role for GGA3 in the pathogenesis of this disorder [33]. Syndromic intellectual disability is characterized by intellectual disability (low intelligence quotient (IQ) and limited adaptive functioning) accompanied by other clinical manifestations. Approximately 50% of intellectual disability cases are estimated to have a genetic basis. Another genetic study identified a monoallelic de novo missense variant (c.296G>A; p.R99H) in *Arf1* that is associated with developmental delay, hypotonia, intellectual disability, and motor stereotypies [132]. In particular, this variant enhanced binding to coat proteins including GGA3, and induced swelling of the Golgi apparatus and recycling endosomal compartment. These findings suggest that the Arf1‐GGA interaction—which regulates protein sorting in the Golgi apparatus and endosomes—is critical for neurodevelopment. As noted previously, murine studies have demonstrated that Gga2 is more critical than Gga1 and Gga3 for embryonic development [95, 96].

The potentially associated of GGAs in human disorders are summarized in Table 3. The precise role of GGAs in the pathogenesis of the aforementioned disorders requires further investigation. Additionally, given that intracellular trafficking pathways are implicated in a broad range of physiological processes and human diseases—including innate immunity [133] and other neurodegenerative diseases [134]—exploring the involvement of GGAs in additional physiological activities and human diseases will be of great significance.

## 10 Conclusion and Perspectives

In conclusion, it is well documented that GGA proteins have versatile roles in multiple intracellular compartments and sorting routes besides their originally identified Golgi association. They capture cargos through the DxxLL motif, ubiquitin tags, and variable conserved domains/sequences, drive the cargos to and from the Golgi, cell surface, endosomes, lysosomes, recycling compartments, and axonal terminals. Thus, they serve as key participants in multiple intracellular sorting pathways, and it is increasingly clear that they have a wide range of biological functions. Moreover, increasing evidence indicates that GGA proteins may be involved in human diseases, through regulating the intracellular sorting of key pathological proteins in these diseases, such as the endocytic degradation, recycling and axonal transport of BACE1 in AD, the endocytic degradation and recycling of EGFR in cancers, and targeting de novo‐synthesized GLUT4 to insulin‐responsive GSCs in T2DM. However, the evidences supporting the roles of GGAs in human diseases, such as AD, cancer and T2DM, are mostly based on preliminary laboratory hints and bioinformatic analysis of genetic and clinical data. This possibility requires much more exploration.

Over all, the biological roles of GGAs and their association with human diseases are increasingly the focus of research attention, and exploring the relevant mechanisms of action may contribute to disease prevention and cure. In this review, we have summarized the well characterized structure basis and sorting functions of GGA proteins, analysed the regulatory mechanisms underlying GGAs' functions as well as their potential association with several human disorders. These may help us understand how GGAs participate in the pathogenesis of human diseases and, in the future, how to intervention the diseases via GGA‐targeted strategies.

## Author Contributions

**Qinqin Wang:** data curation, funding acquisition, writing – original draft. **Juan Mei:** writing – original draft, writing – review and editing. **Shuyue Li:** writing – original draft, writing – review and editing. **Guoan Zhang:** writing – review and editing. **Jian Qiu:** writing – review and editing. **Xuezhi Li:** writing – review and editing, conceptualization, funding acquisition.

## Funding

This work was funded by Natural Science Foundation of Shandong Province (#ZR2022MH244) to X.L., National Natural Science Foundation of China (NSFC) (#82301629), Natural Science Foundation of Shandong Province (#ZR2021QC039) to Q.W.

## Ethics Statement

The authors have nothing to report.

## Consent

The authors have nothing to report.

## Conflicts of Interest

The authors declare no conflicts of interest.

## Data Availability

Data sharing not applicable to this article as no datasets were generated or analysed during the current study.
